# Symptoms and signs in interpreting Gamma-hydroxybutyrate (GHB) intoxication - an explorative study

**DOI:** 10.1186/1757-7241-22-27

**Published:** 2014-04-23

**Authors:** Margareta Warrén Stomberg, Kai Knudsen, Henrik Stomberg, Ingela Skärsäter

**Affiliations:** 1 Gothenburg University, Sahlgrenska Academy, the Institute of Health and Care Sciences, Gothenburg, Sweden; 2 Department of Anaesthesia and Intensive Care, Sahlgrenska University Hospital, Gothenburg, Sweden; 3 Police Department in Örebro County, Örebro, Sweden; 4 School of Health and Social Sciences, Halmstad University, Halmstad, Sweden; 5 University of Gothenburg, Sahlgrenska Academy, Institute of Health and Care Sciences, PO Box 457, SE 405 30 Gothenburg, Sweden

**Keywords:** Gamma-hydroxybutyrate, GHB, Intoxication, Symptom, Sign

## Abstract

**Background:**

Acute poisoning with gamma-hydroxybutyrate (GHB) has been a serious medical and social problem in different parts of the world including Sweden. GHB is a drug of abuse which acts primarily as central nervous system (CNS) depressants. GHB has serious toxicity, although many young users do not recognise GHB as a dangerous drug. The aim of this pilot study was to explore how symptoms with risk of failure in vital functions would be valued among professionals that encounter GHB intoxication in the emergency phase.

**Methods:**

A web-based survey focusing on the assessment of vital clinical signs for possible GHB intoxication using a numeric scale was carried out during April and May 2011. The participants, n 105, are all professionals who encounter GHB intoxicated in the emergency phase, but have different levels of training in GHB intoxication, mainly Registered Nurses (RNs) in southwest Sweden, employed in pre-hospital or emergency departments at somatic and most psychiatric health care facilities, as well as police officers who in their work come into contact with drug users. Responses in the survey were scored according to risk of GHB intoxication with serious failure of vital functions. The score value was then referred to a so-called evidence based priority (EBP) scale and analysed using descriptive statistics and Fisher's exact test.

**Results:**

Cardiac arrest, coma, hypoxia, general convulsions, slow respiratory and heart rate and pale skin are symptoms with the highest risk of serious failure in vital physical functions and were predominantly recognised as such.

**Conclusion:**

Despite the professionals' different levels of training in GHB intoxication, all of them were relatively well aware of and in accordance regarding the most risky symptoms. The interpretation score for the less risky symptoms and signs of GHB intoxication varied depending on their degree of training. The results should be viewed cautiously, as the size of the professional groups and their general knowledge of critical symptoms of GHB poisoning varied.

## Background

During the last 10 years, acute intoxication with gamma-hydroxybutyrate (GHB) has been a serious medical problem in different parts of Europe, Australia, and the USA [1-4]. GHB is a drug of abuse which acts primarily as central nervous system (CNS) depressants.

In Sweden, GHB abuse is primarily centred in the western region. A large number of deaths associated with GHB, as well as co-ingestion with other drugs have been recorded. During 2000 – 2007 twenty-three cases of mortality were attributed to GHB overdose in the western region, ninety-one percent of which had co-ingested other substances. It has also been shown that an overdose of only GHB may lead to death [5,6]. However, there is limited information regarding statistical trends in the world-wide use of GHB and its analogs, with some evidence of higher use in certain geographical areas of Europe [7].

Although GHB in overdose has serious toxicity and may potentially be lethal, many users do not perceive GHB as a dangerous drug, as low doses induce a state of euphoria and increased social abilities. Many also regard it as a natural part of a subculture in which several different drugs are used [8]. However, a danger of using GHB is related to difficulties dosing it correctly, which leads to a high risk for overdose. Furthermore, the most severe effects can occur when GHB is ingested with another depressant, such as alcohol, barbiturates, and Valium [9]. An overdose may lead to several unwanted physiological effects, such as deep unconsciousness, bradycardia, hypothermia, and respiratory depression [1]. GHB is also an endogenous compound in the mammalian brain where it acts primarily as a central nervous system (CNS) depressant. When taken as a recreational drug, common reactions include agitation, restlessness, dizziness, nausea, and visual disturbances. Consumption in higher doses may induce unconsciousness, muscular jerks, involuntary movements, seizures, confusion, aggression, hallucinogenic effects, respiratory depression, and finally coma. In addition, users may experience severe withdrawal symptoms on cessation of the drug, with a serious risk of becoming delirious and requiring intensive care [5]. There is no specific antidote for GHB overdose; treatment is mainly symptomatic with supportive care.

Young people who abuse GHB often exhibit high-risk behaviour, such as driving vehicles under the influence of drugs [10]. Although the police are often the first professionals to encounter intoxicated persons in public places, medical care may be delayed or not initiated, as it can be difficult deciding whether to take the individual to a medical facility or into custody. When a GHB affected person is conscious and cooperative, it is possible that the individual will be taken to a police station rather than a hospital. However, symptoms of intoxication can be delayed and the clinical condition may deteriorate quickly. In cases of severe overdose, the police usually require an ambulance immediately.

Understanding the vital signs of GHB poisoning in the pre-hospital and public setting is important. The dynamic process in the acute phase of drug poisoning in combination with difficulties identifying vital signs make education among those professionals that first encounter GHB abusers in the acute phase essential. There is a lack of scientific studies regarding the levels of knowledge and the priorities required among health care professionals, in order for them to recognise the clinical signs of poisoning [11]. This study may contribute by drawing attention of assessing GHB poisoning and the necessity for prompt medical intervention when required.

The aim of this pilot study was to explore how symptoms indicating risk of failure in vital functions are valued among professionals that encounter GHB intoxication in the emergency phase.

## Methods

A cross sectional web-based survey regarding the assessment of clinical symptoms of GHB intoxication was conducted among health care personnel and police officers.

### Participants

This study was directed towards involving professionals that encounter GHB intoxicated individuals in the emergency phase, those who are “the first on the scene”.

Registered Nurses (RNs) working in pre-hospital care or emergency departments of somatic and psychiatric health care facilities, as well as police officers, were invited to participate. The most informants were chosen because they work in health care clinics in regions of Sweden that have experienced many problems associated with GHB abuse. A convenience sample was selected when the divisional manager of each department, after informed consent, invited the employees to participate, by publishing the link to a web-based questionnaire on the internal website. Employees on duty during a certain week of one month in the survey period April – May 2011 all received web- based information about the study and were given the opportunity to respond to the questionnaire.

However, the Police Authority of the targeted region was unable to publish the link to the web-based survey or as an e-mail questionnaire for their employees. The Authority was subsequently invited to respond to the paper-based questionnaire, but refrained from participating.

Finally, 10 police officers were invited to respond to the same statements paper-based, by the divisional manager in a middle-sized city where GHB was beginning to spread. This paper-based data was then transferred to a SPSS spreadsheet for statistical analysis, together with the other web-based responses (SPSS version 19.0). No ethical approval was needed, according to Swedish ethical guidelines.

### Procedures and data collection

A structured web-based questionnaire was designed, based on the scientific results of several previous clinical studies regarding the signs and symptoms of GHB intoxication [12,13], as well as the medical knowledge of an experienced consultant in clinical toxicology (KK). Statements concerning the clinical signs of GHB intoxication were formulated and graded from 1–8, where 1 indicated no risk that the individual would develop failure of overall physiological function and 8 indicated a pronounced risk of failure or collapse. See Table 1. The statements covered common clinical symptoms of GHB intoxication, including respiratory, circulatory, and neuromuscular symptoms, as well as the degree of mental impairment up to complete loss of consciousness.

**Table 1:** An example of a statement

Clinical signs	The degree of risk that an individual develops failure of overall physiological function, please select a figure
Uncontrolled movements	1 2 3 4 5 6 7 8
	no risk most pronounced risk

Health care informants representing different departments responded to web-based given statements about GHB symptoms and their relevance for GHB intoxication and 10 police officers did so paper-based. One reminder was sent on the internal website of each health care department and a verbal reminder was issued to the manager of the paper-based participants. No personal e-mail reminders were sent. The exact number of participants that were invited to respond is not known, as we do not know how many employees read the invitation on the internal website. However, ten police officers were verbally invited to respond.

### Analysis of collected data

Data gathered from the responses was analysed using descriptive statistics and calculation of Fisher’s exact test. The clinical statements were prioritised according to degree of risk of overall serious failure in vital physical functions due to GHB intoxication, and then referred to as so-called *evidence based priority* (*EBP*). The participants were analysed in two groups; first, those with specific training in GHB intoxication, n 77; pre-hospital RNs and RNs at a general emergency clinic, and second, those participants without specific training in GHB intoxication, n 28; RNs at a psychiatric emergency clinic, not RNs (others) at a general emergency clinic, and police officers who in their work encounter drug intoxicated individuals. See Table 2.

**Table 2:** Demographic data of survey participants

**Profession (n 105)**	**Age mean years (range)**	**Working experience, mean years (range)**	**Sex n males/females**
Prehospital RN (n = 27)	38 (28–55)	10,6 (2–30)	25/2
RN at a general emergency clinic (n = 50)	38,8 (26–60)	9,5 (1–35)	12/38
RN at a psychiatric emergency clinic (n = 6)	48 (31–58)	15 (4–35)	1/5
Police officers (n = 10)	33,9 (30–38)	6,8 (3–10)	8/2
Others (n = 12)	43 (25–60)	15 (6–26)	5/7

The median was calculated for the most pronounced statements regarding intoxication (ranked 6–8, 8 = most pronounced risk) for these two groups. The material was not designed for statistical comparisons between the professional groups.

## Results

We estimate the response rate to be approximately 60% among the web-based participants and 100% for the paper-based respondents (police officers). The study group included 105 participants, with some missing data equally distributed among the statements to be responded to in the questionnaire. Informants from all four health care disciplines responded to the questionnaire, however, general emergency department and pre-hospital nurses were predominant. The police officers had less working experience and they were, as the pre-hospital RNs, mostly males (Table 2).

**Figure 1:**
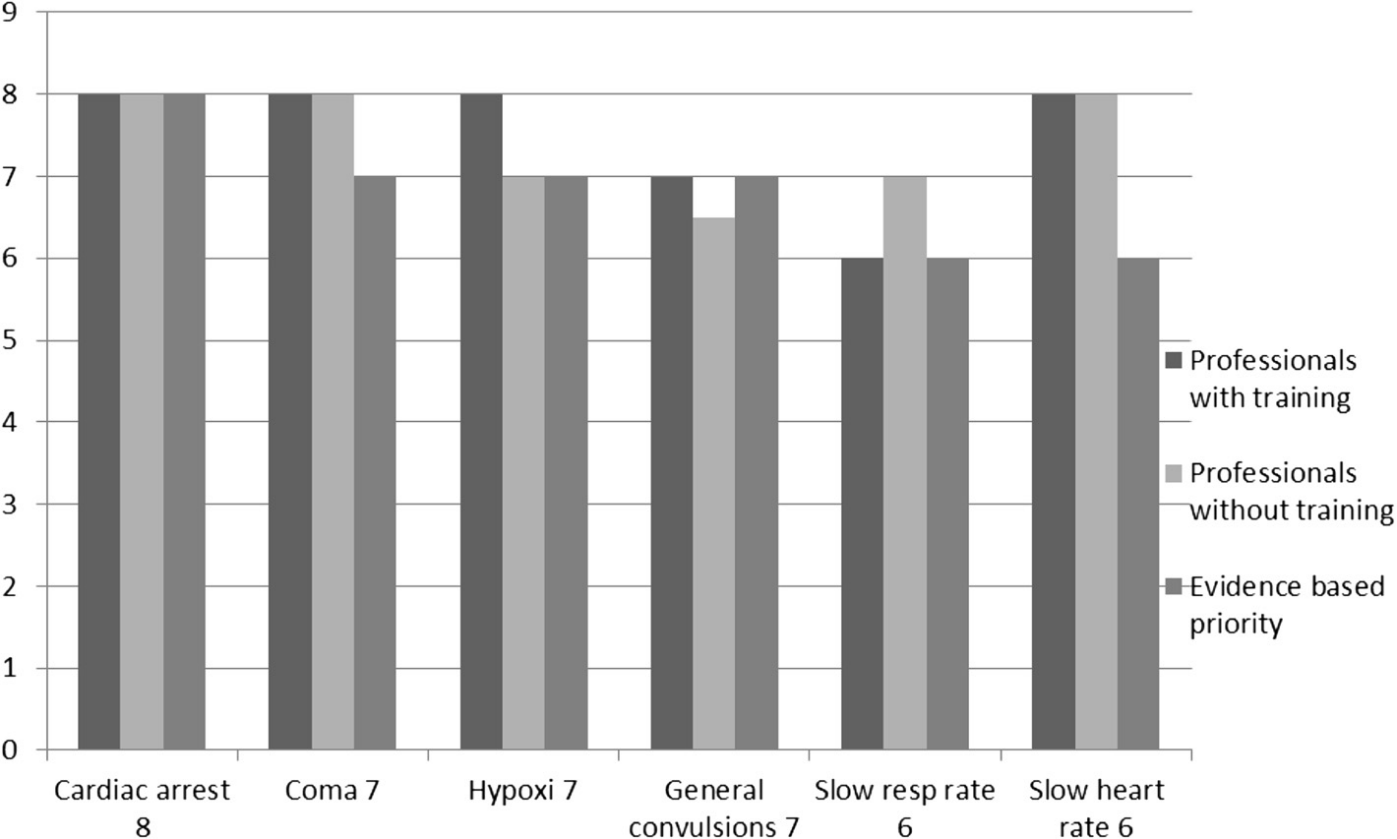
Professionals´ median scoring of GHB intoxication symptoms referring to evidence based priority value, ranked 1–8 (8 = pronounced risk).

Cardiac arrest, coma, hypoxia, general convulsions, slow respiratory and heart rate, as well as pale skin are the most dangerous symptoms of acute GHB poisoning, indicating serious failure in vital physical functions (Figure 1). Although the professionals had different levels of training in GHB intoxication, all of them were well aware of these priorities and identified the symptoms requiring urgent attention. The standard deviations in evaluating signs of GHB intoxication were smaller among the professionals with training in GHB intoxication.

The variations between the groups’ evaluation of the severity of the symptoms as well as between standard deviations were extensive when EBP was ranked <6. All the symptoms were then assessed as more risky in relation to the EBP value, except constricted pupils which were assessed less risky in relation to EBP by both groups. The symptom which was assessed with the most deviation between the groups was hyperactivity. RNs at the emergency department evaluated hyperactivity as a high risk in GHB intoxication, while police officers found it to be a much lower risk symptom. Pale facial skin is the symptom that was judged as significantly more risky in relation to the overall priority, *p* = 0.0011. No significant differences were found in relation to the professionals’ working experiences.

## Discussion

The aim of this study was to explore health care personnel and police officers’ identification of the various clinical risk signs indicating GHB intoxication with possible failure in vital functions. One hundred and five (105) responses from representatives of four different professionals with different working experience may be considered a reliable response to the aim of this study. The consequences of severe GHB overdose with rapid failure in vital function are documented in several studies [2,5,6]. All the participants in this study seemed to be aware of the morbidity and substantial mortality of severe GHB overdose.

The major findings show a relatively consistent evaluation of symptoms with high risk of serious failure in vital organs, despite previous training. However, the evaluations of the less risky symptoms are more varied between the professionals.

Nevertheless, this study should not be regarded as a knowledge inventory of various professionals about how to triage different clinical risk signs of GHB intoxication. The clinical importance of a 5 or 6 score on the scale must be studied further. The difference in the risk evaluations of hyperactivity can be assumed to be explained by the fact that police are often called to stressful emergency incidents during which individuals behave in a hyperactive manner, a situation which is less common in an emergency room. Therefore, police officers do not identify hyperactivity as a specific sign of GHB intoxication. Similarly, police officers often encounter aggressive individuals and do not react specifically to this symptom. However, a GHB intoxicated person may also show paradoxical agitation just before collapse and unconsciousness [14].

Among young people, GHB is usually regarded as a party drug that is often taken on special occasions, so-called all night parties. This is well known among police officers. The circumstances in which police personnel encounter a person influenced by drugs as well as their personal working experience are crucial to raising awareness of the risk of GHB intoxication.

Due to the narrow safety margins and the risk of severe adverse effects associated with GHB and its analogues, it appears that the public, especially young people, needs to be better informed of the risks associated with GHB use [14,15].

Findings from this study indicate the need for professionals who come into contact with a GHB intoxicated individual in the emergency phase to rapidly and safely be able to identify the risk symptoms.

Furthermore, many police officers anticipate a rapid spread of new designer drugs with many unknown or insufficiently recognised physiological effects. The expression of such fears is in accordance with Stein [15] who claims that several websites facilitate the use of GHB and other dangerous drugs. Police authorities have begun collaboration with Centers for Dependency Disorders in our hospital, in order to improve knowledge of GHB and other dangerous party drugs, as well as provide a profile about the inflow of new drugs.

### Study limitations

The correct responses to the statements (referred to as EBP) in the questionnaire were based on earlier studies and the judgment of one experienced physician and cannot be regarded as general consensus or absolutely true.

Since we do not know how many employees saw the link to the questionnaire on the internal website, we cannot accurately calculate the response rate. The police officers responded to the paper-based statements, which may have some impact on the outcome. Therefore, the result cannot be generalised. Further surveys are needed, as much research is based on single hospital case studies of overdose and withdrawal [15].

## Conclusions

The rapid risk evaluation of GHB intoxication symptoms can be lifesaving. Findings in this study indicate that RNs and police officers are all aware of the risk of failure in vital functions with GHB intoxication, and can identify the risk symptoms, although to varying degrees, depending on the level of training in GHB intoxication. Further research is needed to explore the differences between the professionals’ evaluation of the risk symptoms and to identify specific educational needs of the respective professional groups.

## Competing interests

The authors declare no potential conflicts of interest with respect to the research, authorship, and/or publication of this article.

## Authors' contributions

MWS designed the study, collected the data from the RNs, analysed and drafted the manuscript, and wrote the final version. KK contributed to the design of the study, risk evaluation of evidence based priority symptoms and the preparation of the manuscript. HS collected the data from the police officers and contributed to the manuscript with knowledge and future police work associated with drugs. IS contributed to the design and analysis process and preparing the manuscript. All the authors read and approved the final manuscript, as well as the revision.

## References

[B1] Liechti ME Kunz I Greminger P Speich R Kupferschmidt H Clinical features of gamma-hydroxybutyrate and gamma-butyrolactone toxicity and concomitant drug and alcohol use Drug Alcohol Depend 2006 81 3 323 326 10.1016/j.drugalcdep.2005.07.010 16143455

[B2] Miro O Nogue S Espinosa G To-Figueras J Sanchez M Trends in illicit drug emergencies: the emerging role of gamma-hydroxybutyrate J Toxicol Clin Toxicol 2002 40 2 129 135 10.1081/CLT-120004400 12126184

[B3] Wood DM Warren-Gash C Ashraf T Greene SL Shather Z Trivedy C Clarke S Ramsey J Holt DW Dargan PI Medical and legal confusion surrounding gamma-hydroxybutyrate (GHB) and its precursors gamma-butyrolactone (GBL) and 1,4-butanediol (1,4BD) QJM 2008 101 1 23 29 18203723 10.1093/qjmed/hcm117

[B4] Zvosec DL Smith SW Porrata T Strobl AQ Dyer JE Case series of 226 gamma-hydroxybutyrate-associated deaths: lethal toxicity and trauma Am J Emerg Med 2011 29 3 319 332 10.1016/j.ajem.2009.11.008 20825811

[B5] Knudsen K Greter J Verdicchio M High mortality rates among GHB abusers in Western Sweden Clin Toxicol (Phila) 2008 46 3 187 192 10.1080/15563650701263633 18344100

[B6] Knudsen K Jonsson U Abrahamsson J Twenty-three deaths with gamma-hydroxybutyrate overdose in western Sweden between 2000 and 2007 Acta Anaesthesiol Scand 2010 54 8 987 992 10.1111/j.1399-6576.2010.02278.x 20701597

[B7] Schep LJ Knudsen K Slaughter RJ Vale JA Megarbane B The clinical toxicology of gamma-hydroxybutyrate, gamma-butyrolactone and 1,4-butanediol Clin Toxicol (Phila) 2012 50 6 458 470 10.3109/15563650.2012.702218 22746383

[B8] Barker JC Karsoho H Hazardous use of gamma hydroxybutyrate: driving under the influence Subst Use Misuse 2008 43 11 1507 1520 10.1080/10826080802237928 18752156

[B9] Galicia M Nogue S Miro O Liquid ecstasy intoxication: clinical features of 505 consecutive emergency department patients Emerg Med J 2011 28 6 462 466 10.1136/emj.2008.068403 21602168

[B10] Kim SY Anderson IB Dyer JE Barker JC Blanc PD High-risk behaviors and hospitalizations among gamma hydroxybutyrate (GHB) users Am J Drug Alcohol Abuse 2007 33 3 429 438 10.1080/00952990701312316 17613970 PMC2257866

[B11] Folkhälsoinstitut S Balancing on the border of consciousness GHB and closely related compounds – Risk use, abuse and dependence – a knowledge inventory Swe: Balansering på medvetandets gräns. Risk, missbruk och beroende av GHB och närbesläktade preparat – en kunskapsinventering 2011 Stockholm: Institute of Public Health

[B12] Couper F Marinetti L Gamma-Hydroxybutyrate (GHB) – effects on human performance and behavior Forensic Sci Rev 2002 14 12 101 122 26256488

[B13] Jones AW Holmgren A Kugelberg FC Driving under the influence of gamma-hydroxybutyrate (GHB) Forensic Sci Med Pathol 2008 4 4 205 211 10.1007/s12024-008-9040-1 19291440

[B14] Drasbek KR Christensen J Jensen K Gamma-hydroxybutyrate–a drug of abuse Acta Neurol Scand 2006 114 3 145 156 10.1111/j.1600-0404.2006.00712.x 16911342

[B15] Stein LA Lebeau R Clair M Martin R Bryant M Storti S Monti P A web-based study of gamma hydroxybutyrate (GHB): patterns, experiences, and functions of use Am J Addict 2011 20 1 30 39 10.1111/j.1521-0391.2010.00099.x 21175918 PMC3065821

